# Nucleolar expansion: A biomolecular condensate mortality timer

**DOI:** 10.70401/geromedicine.2026.0017

**Published:** 2026-03-11

**Authors:** J. Ignacio Gutierrez, Jessica K. Tyler

**Affiliations:** Weill Cornell Medicine, Department of Pathology and Laboratory Medicine, New York, NY 10065, USA.

**Keywords:** Nucleolus, aging, longevity, condensates, lifespan extension, mortality timer

## Abstract

The nucleolus, the largest membraneless organelle in the cell, is a biomolecular condensate that houses ribosomal DNA (rDNA), facilitates ribosomal subunit assembly, and serves as a dynamic reservoir for numerous unrelated proteins. Aging across eukaryotic species is accompanied by nucleolar expansion, raising the question of whether it is a correlate of aging or a driver of cellular aging. Recent studies suggest that nucleolar expansion may drive aging and this may result from age-associated changes in the biophysical properties of the nucleolus. Emerging evidence points to age-driven biophysical changes in the nucleolar condensate, including shifts in size, dynamics, and viscoelasticity, which may occur gradually or through transitions from a liquid-like state to denser gel-like, and in some contexts amyloid-like, assemblies. These transitions remodel two core condensate properties: compartmentalization and partitioning, with consequences for ribosome biogenesis and rDNA stability. Here, we review recent literature on age-driven changes in nucleolar condensation and discuss how these changes may influence nucleolar function and longevity.

## 1. Introduction

Most interventions known to enhance longevity in mammals are closely linked to cellular growth and metabolism (extensively reviewed in previous studies^[1,2]^). For instance, dietary restriction and reduced insulin/IGF-1 signaling extend lifespan by suppressing growth-promoting pathways and enhancing stress resistance^[1,3]^. Similarly, inhibition of mTOR/Tor1, either genetically or pharmacologically, extends lifespan from mammals to yeast by activating autophagy, and concurrently reduces protein synthesis^[4]^. Activation of AMPK, a central energy sensor that promotes catabolic over anabolic processes, also enhances longevity by improving metabolic efficiency and stress tolerance^[5]^. Together, these findings suggest that lifespan extension can be achieved by reprogramming cellular priorities from biosynthesis and proliferation toward stress resilience and reduced anabolic demand. Ribosome biogenesis is a major consumer of biosynthetic resources, and ribosome abundance itself determines translational capacity and consequently, metabolic flux.

The nucleolus, where ribosomal DNA (rDNA) is transcribed into ribosomal RNA (rRNA) and further processed into ribosomal subunits, sits at the heart of this growth–metabolism axis^[6]^. Historically, nucleolar expansion was among the first recognized cellular phenotypes of cellular aging^[7]^, although the underlying reason was unclear. Coherently, nucleolar size decreases in response to several lifespan-extending regimens, such as reduced insulin/IGF signaling, mTOR inhibition and dietary restriction, providing a common morphological signature of pro-longevity physiology^[8]^. Yet it remained unresolved whether the small nucleolar size observed under pro-longevity regimens merely mirrors metabolic reprogramming or can *actively* drive anti-aging. Our recent work supports the latter view^[9]^, where the increase in nucleolus size and accompanied rDNA instability during aging act as a countdown to cell death, i.e., as a mortality timer. Nucleolar size has long served as a proxy for ribosome biogenesis output, where larger nucleoli correlate with greater ribosome biogenesis^[8]^. As phase-separated condensates, nucleoli exhibit changes in size or number that often reflect underlying shifts in biophysical properties, such as liquidity, viscosity, or fusion behavior^[10,11]^, which can influence nucleolar function during aging^[8,9]^. In this review, we discuss how these nucleolar variables change with age and whether they, in turn, influence longevity.

Historically, the nucleolus has been recognized since the late 18th century: Felice Fontana sketched it in 1781, well before ‘organelle’ entered the cell-biology lexicon. Only recently, however, have its multidimensional functions begun to be elucidated. Unlike membrane-bound organelles, the nucleolus lacks a delimiting membrane, forms through condensation of nucleic acids and proteins, and scaffolds into coexisting subcompartments, providing *compartmentalization* of nucleolar processes. Selective *partitioning* of proteins and RNAs across these subcompartments creates a specialized chemical and physical environment optimized not only for ribosomal subunit assembly but also for housing and maintenance of the fragile, repetitive rDNA, which encodes the RNA components of ribosomes^[12]^. Notably, the nucleolus also selectively recruits proteins not dedicated to ribosome biogenesis or rDNA related (e.g., stress response and cell-cycle regulators)^[13]^, implying cellular roles that extend beyond ribosomal subunit production. How aging-associated nucleolar expansion and changes in its condensation properties perturb this selective nucleolar partitioning and how these impact genome maintenance and stress signaling, are central questions addressed in this review.

## 2. The Nucleolus is A Biomolecular Condensate

**Figure 1.**
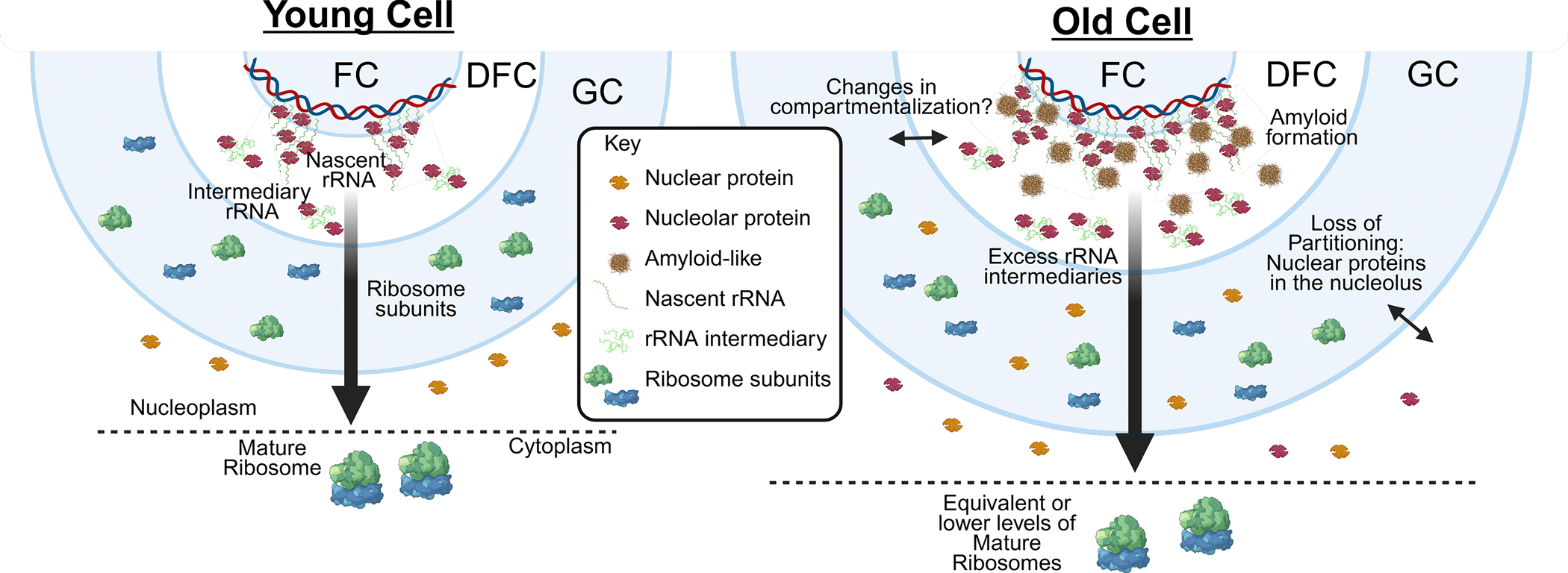
Model for age-associated changes in nucleolar organization. In old cells, rDNA transcription increases while ribosome biogenesis remains unchanged, leading to an accumulation of rRNA intermediates. This imbalance may alter the biophysical properties of the nucleolus, including the appearance of amyloid-like structures in the FC/DFC and reduced partitioning fidelity, allowing nuclear proteins to aberrantly accumulate within the nucleolus. These changes may also affect nucleolar compartmentalization during aging, although this possibility remains to be demonstrated. rDNA: ribosomal DNA; rRNA: ribosomal RNA; FC: fibrillar center; DFC: dense fibrillar component; GC: granular component.

From the earliest light micrographs, reviewed by Thomas H. Montgomery in 1898, the nucleolus was described as a darker, distinct body within the nucleus, providing an early indication of nuclear compartmentalization^[14]^. Classic cytochemistry and extraction experiments then suggested a matrix- or gel-like structure, as removal of RNA by ribonuclease treatment and partial pepsin digestion left a stable fibrillar meshwork of proteins and DNA^[15]^. However, a landmark study in Xenopus laevis eggs from Tony Hyman’s lab provided the first direct biophysical evidence that nucleoli behave as phase-separated liquid droplets that fuse and flow with viscosity and surface tension^[ 10]^. Importantly, the nucleolus is not a homogeneous liquid structure but contains coexisting subcompartments^[16]^, two in budding yeast and Caenorhabditis elegans, and three in vertebrates. The three nucleolar subcompartments are the fibrillar center (FC), dense fibrillar component (DFC), and granular component (GC). These subcompartments are arranged in a nested structure, with DFC surrounding the FC, and multiple FC/DFC cores embedded within the larger GC subcompartment (Figure 1).

The nucleolar subcompartments form through self-assembly into emulsion-type subphases, driven by the biochemical properties of their constituent proteins. *In vitro* reconstitution experiments reveal that recombinant nucleolar scaffold proteins such as nucleophosmin (NPM1, found in the GC) and fibrillarin (FIB1, found in the DFC), can independently undergo phase separation, recreating distinct nucleolar subcompartments when mixed *in vitro*^[16–18]^. Interestingly, siRNA knock-down of NPM1^[19]^, FIB1^[20]^ and other nucleolar proteins alters nucleolar morphology and affects ribosome biogenesis, but does not prevent nucleolar formation, indicating that such formation relies on redundant or overlapping scaffolding mechanisms. Both NPM1 and FIB1 contain glycine-arginine-rich (GAR) domains that drive multivalent interactions, facilitating liquid–liquid phase separation and promoting the formation of multiphase condensates^[16–18]^. These GAR domains constitute a defining feature of many nucleolar proteins^[21]^ and are crucial for nucleolar assembly, as well as for the unique viscoelastic and emulsion-like properties of these proteins observed in cells^[16,17]^. However, the fine-tuning of nucleolar density and viscoelasticity is governed by the organelle’s central function, ribosomal subunit biogenesis^[11,22]^. rDNA transcription occurs at the interface between the FC and DFC, a densely crowded environment where nascent rRNA is coated by pre-rRNA processing proteins, increasing local density^[11]^. As ribosomal subunit assembly proceeds, the interactions with multivalent pre-rRNA processing and ribosome assembly factors diminish, generating a decreasing gradient of subcompartment density^[23]^. This gradient streamlines ribosome subunit maturation and supports the unidirectional flow necessary for efficient ribosome subunit biogenesis, as demonstrated by recent findings in both live cells and *in vitro* studies^[22,24]^.

## 3. Nucleolar Expansion During Aging

In the late 1970s, Bemiller & Lee observed that nucleolar size increases dramatically in old senescent cultured human fibroblasts, with multiple smaller nucleoli often coalescing into a single, enlarged structure, a phenomenon subsequently confirmed in other aged organisms, including yeast and worms^[7,25,26]^ (Figure 1). In yeast, deletion of *SGS1*, the homolog of the human WRN gene whose mutation causes Werner’s syndrome, a premature aging disorder, results in abrupt nucleolar expansion and fragmentation during aging, accompanied by a reduction in replicative lifespan (i.e., the number of cell divisions in a cell’s lifespan)^[27]^. Consistently, the nucleoli from fibroblasts isolated from humans of different ages show increased size with age, while fibroblasts from young patients with Hutchinson Gilmore Progeria Syndrome, another premature aging disorder, display enlarged nucleoli^[26]^. Supporting the idea that nucleolar enlargement can reflect a pathogenic nucleolar stress state, systemic induction of nucleolar stress in mice through expression of arginine-rich poly(PR) peptides increases fibrillarin-positive nucleolar area across tissues and accelerates organismal aging, leading to premature death^[28]^. Conversely, small nucleolar size is induced by numerous pro-longevity regimens^[29]^. Reduction of nucleolar size is also observed during gametogenesis-mediated cellular rejuvenation in yeast^[30]^, and during *C. elegans* lifespan extension caused by RNAi depletion of the nucleolar protein fibrillarin^[8]^. Strikingly, in *C. elegans*, nucleolar size in hypodermal cells measured on the first day of adulthood inversely correlates with remaining lifespan, further strengthening the negative correlation between nucleolar size and longevity across taxa^[8]^.

In young cells, nucleolar size is closely linked to rDNA transcription^[31,32]^. Elevated rDNA transcription drives increased ribosomal subunit biogenesis, accompanied by nucleolar enlargement. Size is a highly dynamic feature of biomolecular condensates^[18,24]^, and modulation of the nucleolar size in response to rRNA synthesis could help maintain constant molecular crowding and biophysical properties to allow efficient ribosome assembly. Indeed, in aged human fibroblasts and fibroblasts from young progeria patients, nucleolar expansion is associated with increased ribosome biogenesis and an overall increase in protein synthesis^[26]^. However, this is not the case in other cell types and organisms because in yeast, age-driven nucleolar expansion occurs without increased ribosome biogenesis^[8,9,33]^. In multiple human cell types, including neurons, hepatocytes, and muscle cells, aging is accompanied by a modest decline in ribosome abundance and translational efficiency^[34,35]^. This raises the possibility that age-associated dissociation between nucleolar size and ribosome biogenesis may contribute to the detrimental impact of nucleolar expansion on longevity (Figure 1). A non-aging pathological example of such imbalance between nucleolar size and ribosome biogenesis is observed in spinal muscular atrophy (SMA) motor neurons, where nucleoli become markedly enlarged and the FC subcompartment expands while decreasing in number. Notably, SMA neurons also show altered rRNA homeostasis, including accumulation of rRNA species, supporting the idea that nucleolar hypertrophy can reflect stalled or dysregulated ribosome production rather than elevated biosynthetic activity^[36]^. Consistent with this idea, aging brain proteomics reveals loss of ribosome stoichiometry and accumulation of orphan ribosomal proteins in aggregates, suggesting that impaired ribosome assembly and proteostasis collapse can decouple nucleolar activity from functional ribosome output^[37,38]^.

During aging, it is not yet clear whether the size of all nucleolar subcompartments is enlarged or whether only specific subcompartments are affected. Clarifying this distinction could provide important insight into the molecular reason for their enlargement. For example, preferential enlargement of the GC subcompartment may indicate impaired nucleolar export of pre-ribosomal particles^[39,40]^.

**Figure 2.**
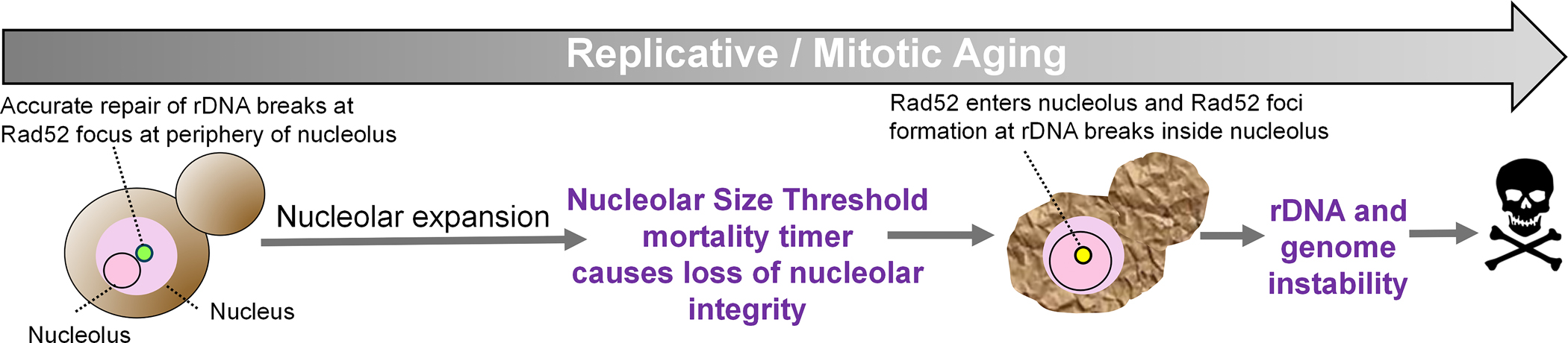
Nucleolar size threshold as an irreversible transition during yeast replicative aging that causes rDNA instability and ends the lifespan. In young cells, and for most of the replicative lifespan, the nucleolus remains small and repair of rDNA DSBs is restricted to the nucleolar periphery. As cells age, nucleolar expansion accelerates abruptly upon approaching the NST. Once the NST is exceeded, nucleolar integrity is lost and Rad52 enters the nucleolus, forming repair foci at rDNA breaks within the nucleolar compartment, triggering inaccurate rDNA recombination, associated global genome instability and imminent cell death, consistent with the NST acting as a mortality timer. rDNA: ribosomal DNA; DSBs: DNA double-strand breaks; NST: nucleolar size threshold.

To distinguish whether nuclear expansion is a cause or a consequence of aging, we engineered yeast to express dCas9 fused to the inner nuclear membrane protein Heh1, guided by RNAs targeting the rDNA^[9]^. This rDNA-tethering system delayed expansion of the yeast nucleolus during aging, resulting in lifespan extension^[9]^. Even in wild-type cells, the yeast that expanded their nucleolus later during aging, lived longer^[9]^. Thus, while nucleolar expansion occurs as a consequence of aging, it clearly limits cellular longevity. Furthermore, live cell imaging of the GFP-tagged yeast nucleolus throughout the lifespan of yeast trapped in microfluidic devices revealed an unexpected finding. Although nucleolar expansion progresses gradually during aging, once a critical size termed the Nucleolar Size Threshold is reached, subsequent expansion accelerates markedly^[9]^. Strikingly, after the nucleolar size threshold was surpassed, the cells divided on average only five more times before death. Together, these findings suggest that nucleolar expansion, upon reaching a certain threshold, marks a transition to a new biophysical state that functions as a mortality timer during aging (Figure 2), setting the stage for understanding how rDNA instability emerges as a downstream consequence.

## 4. rDNA Instability and Age-Driven Nucleolar Expansion

DNA double-strand breaks (DSBs) within the rDNA occur naturally across organisms from yeast to humans, arising from a roadblock mechanism that prevents collisions between the DNA replication and transcription machinery^[41]^. However, rDNA instability greatly increases with age, as observed in yeast^[42,43]^, *Drosophila*^[44,45]^, mice^[46]^ and human aging^[47]^, as well as in age-related disease^[48]^ and progeroid contexts^[49]^. rDNA instability can be easily detected during yeast aging^[43]^, due to the presence of a single rDNA locus that can be excised from the genome and resolved by pulsed-field electrophoresis to detect gains and losses of rDNA repeats, and by the accumulation of extrachromosomal rDNA circles (ERC) evicted from the genome as a consequence of intrachromosomal rDNA recombination^[42]^. In addition, the rDNA locus in aged yeast was identified as a hotspot of gammaH2A (the yeast equivalent of the gammaH2AX marker of DNA DSBs) accumulation in ChIP-seq analyses, a frequent translocation partner in age-induced chromosomal translocations, and a site of recurrent amplification of the chromosomal region distal to the rDNA locus^[50]^. By contrast, detection of rDNA instability during aging in mammals remains technically challenging because the rDNA repeats are distributed across multiple acrocentric chromosomes, are highly homogeneous, and are poorly resolved by standard genomic and cytological approaches^[49]^. Extrachromosomal circular DNA (eccDNA) has been reported to increase with cellular aging in mammalian systems^[51]^. In humans, eccDNAs have been shown to include circles derived from rDNA sequences, specifically 5S rDNA^[52]^, and other rDNA-origin eccDNAs detected in somatic tissues^[51]^. Nevertheless, it remains unknown whether rDNA-derived eccDNAs accumulate as part of normal human physiological aging. Using an advanced live-cell imaging approach (thioredoxin-fused TALE live-imaging system), a reduction in rDNA repeat number has been observed in human mesenchymal stem cells in blood from elderly individuals and progeroid patients^[47]^. Meanwhile, deletion of the gene encoding sirtuin SIRT7 from human fibroblasts impaired heterochromatin silencing at rDNA, leading to rDNA copy-number loss and subsequent induction of senescence^[53,54]^.

We recently uncovered that age-driven nucleolar expansion in yeast triggers rDNA instability due to mislocalization of the DNA repair machinery (Figure 2). DNA DSBs within the rDNA are normally repaired by homologous recombination at the nucleolar periphery to favor use of the corresponding undamaged rDNA repeat from the sister chromatid as the homology template. This deters aberrant recombination using one of the ~150 other rDNA repeats as the homology template, which may occur if homologous recombination took place within the nucleolus in yeast^[55]^. Homologous recombination of rDNA breaks is limited to the nucleolar periphery as the DNA DSB relocate to this region^[55]^. While in mammalian cells, rDNA DSBs trigger the formation of a nucleolar cap at the periphery, a specialized repair compartment comprising rDNA with FC/DFC material^[56]^. In yeast, homologous recombinational repair of rDNA DSBs is restricted to the nucleolar periphery because Rad52, the yeast functional equivalent of mammalian Brca2, is actively expelled from the nucleolus, allowing repair to proceed only after the rDNA DSBs exit the nucleolus^[55]^. Unexpectedly, we observed that in old yeast with nucleoli exceeding the nucleolar size threshold, Rad52-GFP foci appeared within the nucleolus rather than at the periphery, triggering rDNA hyperrecombination and imminent death^[9]^ (Figure 2).

In old yeast lacking Rad52 foci, free Rad52 and another normally nucleolar-excluded protein were observed to enter the expanded nucleoli^[9]^. Furthermore, a large nucleolus is sufficient for Rad52 entry, given that expansion of nucleoli in young yeast, induced by treatment with the Sirtuin inhibitor resveratrol, similarly resulted in Rad52-GFP foci within the nucleoli^[9]^. Notably, an engineered system that delays yeast nucleolar expansion during aging resulted in reduced rDNA instability, as detected by ERC formation and lifespan extension^[9]^, highlighting the relationship between rDNA instability and nucleolar size. Interestingly, recent work in post-mitotic mammalian neurons demonstrated that Rad52 recruitment to DNA DSBs can be transcription-dependent and requires RNA–DNA hybrid structures (R-loops), supporting a conserved role for Rad52 in RNA-templated recombinational repair when canonical sister-chromatid templates are unavailable. This suggests that nucleolar exclusion of Rad52 may not only spatially restrict recombination but also prevent inappropriate engagement of transcription-associated, RNA-templated repair pathways within the rDNA-rich nucleolar environment^[57]^. These studies collectively indicate that aging-driven nucleolar expansion leads to rDNA instability not solely due to the increased size *per se*, but also owing to changes in other biophysical properties of the nucleolus, which may be reflected in its changed size, thereby affecting its function and tilting the system toward rDNA instability.

Importantly, age-induced rDNA instability also promotes global genome instability. This was evident in the reduced chromosomal fragmentation of yeast deleted for *FOB1*, the roadblock protein that induces the rDNA breaks^[43]^. Furthermore, engineering the yeast nucleolus to remain smaller during aging both reduced and delayed the appearance of Rad52-GFP foci, reflecting sites of homologous recombinational repair^[9]^. Exactly why rDNA instability during aging induces genome-wide instability is not clear, but it may reflect competition for limited pools of DNA repair factors^[43]^. Regardless, these findings underscore that nucleolar size has implications beyond rDNA instability alone, presumably contributing to cellular death. What is driving the changes in the nucleolar condensate that correlate with, and potentially cause, nucleolar expansion, leading to age-induced rDNA instability and end of lifespan?

## 5. Changes in the Condensation Properties of the Nucleolus

Biomolecular condensates span a continuum of material states; their components can be diluted in solution, or phase-separate into liquid, gel-like, or solid amyloid-like structures. Small environmental or chemical changes, such as variations in pH, ions, RNA/protein stoichiometry and post-translational modifications, can shift them along this spectrum in a highly dynamic way^[58,59]^. The metazoan nucleolus provides a well-established example of condensate dynamics. It disassembles at mitosis and reassembles in G_1_^[60]^, and under nucleolar stress it can densify and undergo gelation-like transitions. For instance, RNA polymerase II (Pol II) transcription at rDNA intergenic spacers (IGS) generates protective RNA-DNA hybrids in the form of R-loops, which suppress disruptive IGS sense transcripts (sincRNAs) and thereby support RNA polymerase I (Pol I)-driven rRNA synthesis^[61]^. Acute Pol II inhibition releases the sincRNAs and reorganizes the nucleolus, causing the formerly nucleolar space to become Congo-red–positive, consistent with an amyloid-like state^[61]^. During heat shock, stress-induced IGS lncRNAs remodel the nucleolus, and sustained stress produce reversible amyloid-like “A-bodies” within the nucleolus that are Congo-red and ThT-positive^[62,63]^. Similarly, proteotoxic stress pushes the nucleolus, especially the GC, into a denser, more elastic/gel-like state with slower FRAP, and sustained stress can further drive the formation of amyloid-like structures such as A-body^[13]^.

## 6. Age-Driven Changes in Nucleolar Condensation

The idea that aberrant phase transitions of condensates could be a driver of cellular aging has been hypothesized^[64]^, but direct experimental evidence for this mechanism has recently begun to emerge. Across species, nucleolar aging has been associated with densification, i.e., transitioning from a liquid-like state to a gel and in some cases, an amyloid-like state. In intestinal epithelial cells of C. elegans, FIB1-GFP (fibrillarin, localized at the rDNA transcription site DFC) shows slower FRAP recovery in old adults than in larvae, indicating reduced molecular exchange within the rRNA-processing layer and a shift toward a more gel-like nucleolar state^[16]^. Notably, *in vitro* reconstitution of fibrillarin-rich droplets reveals time-dependent “maturation”, slower fusion and rounding and diminished internal rearrangements, coherent with the *in vivo* slower FRAP recovery^[16]^. Yeast replicative aging experiments, which model the functional decline accompanying successive cell divisions, show that expanded nucleolus beyond a critical size threshold becomes resistant to 1,6 hexanediol dissolution^[9]^. Importantly, resistance to 1,6 hexanediol (as well as reduced FRAP recovery) indicates decreased condensate dynamics, but does not constitute definitive evidence for cross-β amyloid fibril formation^[65,66]^.

In human cell systems, cellular senescence provides an experimentally tractable model for cellular aging, i.e., a stable cell growth arrest after a finite number of divisions^[67]^, triggered by telomere attrition and/or DNA-damage. Like yeast replicative aging, it culminates in loss of proliferative capacity and features nucleolar expansion^[7,25,26,67]^. The accumulation of senescent cells is a hallmark of aging^[68]^. Several cell types undergo senescence induced by a group of agents that reduce genome integrity by causing DNA lesions/replication stress, and by agents that disrupt gene expression programs (rDNA/Pol I, Pol II, epigenetic modifiers); additional drug classes (CDK4/6 inhibitors, proteostasis stressors) also drive senescence via non-genotoxic routes^[69]^. Importantly, senescence induced by Pol I inhibition^[70]^, CDK4 inhibition^[71]^, oxidative stress^[72]^ and DNA damage^[72]^, although not identical to replicative senescence^[69]^, conserves the expanded nucleolus phenotype^[72,73]^. Under multiple *in vitro* stress-induced senescence models, the density of the DFC subcompartment of the nucleolus increases, as measured by FIB1-GFP FRAP^[72]^, similarly to that observed in C. elegans^[16]^. Moreover, super resolution imaging shows the appearance of fibrils stained by Congo red strongly suggesting the appearance of amyloid-like features in the nucleolus^[72]^. Similarly, another study using label-free vibrational spectroscopy in binucleation-driven senescence identified β-sheet–like, aggregation-prone signatures within nucleoli, providing molecular evidence for nucleolar solidification^[73]^.

Changes in the phase behavior of proteins with low-complexity domains have been extensively studied in the context of neurodegeneration and age-associated neurological disorders, particularly with respect to the formation of amyloid-like aggregates (reviewed in the work^[74]^). A recent study reported that amyloid-positive nucleolar “cavities” are prevalent in postmortem human neurons from aged brains, including neurologically healthy individuals and patients with neurodegenerative diseases. These cavities are intranucleolar inclusions that exclude canonical nucleolar markers such as NPM1, FIB1 and PolI subunit RPA194, but stain positively with Congo red, consistent with amyloid-like material^[75]^. While these structures were common in aged neurons across conditions, neurodegenerative disease was associated with the selective sequestration of specific aggregation-prone proteins within the nucleolus, including phosphorylated tau and TDP-43. Notably, rDNA instability has previously been linked to neurodegenerative diseases^[48]^, raising the possibility that age-associated changes in nucleolar condensation may contribute to genomic instability at rDNA loci, although a causal relationship remains to be established. Collectively, these studies suggest that across taxa aging-associated changes in the nucleolus lead to a denser, gel-like, or even aggregated nucleolus, particularly at its inner layer where nascent rRNA starts folding with the ribosomal proteins (Figure 1).

What drives the denser nucleolar phenotype during aging remains unclear; however, one possible explanation could be a reported increase in PolI-mediated rRNA production^[26,33]^ that is not accompanied by increases in ribosome biogenesis^[9,33]^, creating “traffic” in the unidirectional highway of ribosome biogenesis within the nucleolus. Indeed, perturbations that stall early pre-rRNA processing slow FRAP recoveries, indicating increased density/crowding at the inner core of the nucleolus^[22]^, whereas inhibition of PolI leads to faster FRAP recoveries^[76]^. Collectively, these findings suggest a conserved biophysical alteration in aging cells where stress-induced senescence and dysregulated rRNA flux coincide with the transformation of the nucleolus into a dense, aggregation-prone compartment, potentially disrupting the spatial organization and partitioning of nucleolar proteins (Figure 1). Whether this phenotype is fully recapitulated during replication-induced senescence remains to be determined.

## 7. Nucleolar Partitioning and Compartmentalization

Condensation (phase separation) propensity and partitioning are not identical, although they often correlate because both depend on the same multivalent interactions^[59,77]^. Partitioning refers to the thermodynamic preference to reside in the nucleolar network (partition coefficient: nucleolus/nucleoplasm); phase separation describes the emergent formation of a dense biomolecular phase coexisting with a surrounding dilute phase^[11,59]^. Thus, a mutation can in principle lower phase separation while maintaining or even raising partitioning (e.g., by strengthening specific heterotypic binding), or reduce partitioning without abolishing phase separation (e.g., if stoichiometry becomes limiting)^[17,78]^. As mentioned above, nucleolar condensation relies on multiple overlapping scaffolding mechanisms. Mutations targeting the acidic A-tracts and basic B-tracts of NPM1, charged sequence motifs within its intrinsically disordered region that mediate multivalent interactions with rRNA and protein partners^[17]^, provide a concrete example, at least for NPM1, for assessing the relationship between condensation and partitioning of the nucleolus. *In vitro*/*in vivo* comparisons show clear concordance for this set of NPM1 mutants. NMP1 mutA3 lowers the threshold for rRNA-driven phase separation^[17,22]^ and, in cells, exhibits higher partitioning with slower FRAP (stronger retention)^[22]^. In contrast, NMP1 mutB2 (and to a lesser extent NMP1 mutB1) reduces phase separation capacity *in vitro*^[17]^ and, in cells, lowers partitioning with faster FRAP, severely for B2 and modestly for B1^[22]^. Therefore, mutations that make NMP1 more prone to phase separate *in vitro* show increased partitioning (or retention) of NMP1 in the nucleolus, and on the other hand, mutants that reduce the ability to phase separate *in vitro* show lower partitioning (or retention) in the nucleolus^[17,22]^. Extending this logic, one may hypothesize that age-driven shifts toward slower FRAP and nucleolar amyloid-like signatures may reflect increased network connectivity that alters partitioning rules, for instance: whether a protein enters, is retained within, or excluded from the nucleolus. In parallel, mesoscale compartmentalization is rewired: subphase boundaries blur and coarsen (fewer but larger bodies; a denser inner layer), eroding the layered architecture that normally orders rRNA processing and repair routing. In our work, expanded nucleoli in aged cells lose selectivity and nuclear partitioning, admitting entry to proteins normally excluded from small, young nucleoli (e.g., Rad52 and inert GFP), with functional costs for genome maintenance^[9]^. Consistent with this idea, Na-butyrate–induced senescent human cells revealed selective protein relocalization: NPM1 was depleted from nucleoli (accumulating in nucleoplasm), while PARP1 and DHX9 (non-nucleolar proteins) became enriched in nucleoli^[79]^. This phenotype was observed in the absence of shutdown of rRNA synthesis, hinting at changes in partitioning and compartmentalization^[79]^. Although the age-driven changes in nucleolar condensate properties that erode selectivity and permit entry of normally excluded nuclear proteins remain to be determined, several *in vitro* and engineered condensate studies provide clues regarding which parameters may be altered. In particular, RNA concentration and RNA:protein stoichiometry strongly influence condensate architecture and client partitioning^[80,81]^, which would be relevant if age-driven nucleolar expansion involves accumulation of pre-rRNAs. In parallel, increased scaffold self-association and enhanced network connectivity can drive multiphase organization and percolated, gel-like states that alter permeability and retention^[82–84]^, in the scenario that scaffolding proteins required for nucleolar phase separation may have altered abundance and/or interaction valency with age. Finally, recent work suggests that condensates can develop mesh-like internal structures with tunable accessibility, raising the possibility that dense inclusions could disrupt nucleolar compartmentalization and protein routing^[85,86]^. Together, these studies highlight that condensate permeability and viscoelasticity are highly sensitive to condensate composition. Thus, although nucleolar assembly is generally robust and supported by redundant scaffolding systems, age-driven shifts in RNA/protein stoichiometry or scaffold connectivity may ultimately overwhelm this resilience and compromise nucleolar homeostasis.

## 8. Concluding Remarks and Perspectives

Deciphering the mechanisms of aging has become a high priority in the context of a rapidly aging global population. To provide a conceptual structure for the expanding field of aging research, the “hallmarks of aging” framework was proposed, defining a dozen core processes that contribute to age-associated decline^[1]^. The nucleolus directly or indirectly intersects with most of these hallmarks through its central role in ribosome biogenesis and additional regulatory functions. Moreover, the repetitive nature and extremely high transcriptional activity of rDNA, necessary to meet cellular ribosomal demand, render it particularly vulnerable to genomic instability, itself a hallmark of aging^[1]^. The nucleolus also integrates signals from numerous well-studied pro-longevity interventions^[29]^, modulating its architecture in response.

Recent studies have highlighted that the biophysical properties of the nucleolus, including its material state, internal compartmentalization, and the partitioning of the nuclear/nucleolar proteins, are critical for the precision and robustness of its functions. Although nucleolar size does not directly reflect the condensation state of the nucleolus, it is a readily quantifiable variable that often correlates with altered nucleolar properties during aging^[8,9,16,26,72]^. Increasing evidence suggests that aberrant phase transitions may contribute to, rather than merely accompany, the aging process^[9,16,72]^.

It is obvious that the changes in the nucleolar condensate are driving rDNA instability and limit lifespan in budding yeast mitotic aging^[9]^, whether nucleolar expansion acts as a driver of organismal aging in mammals remains unresolved and will require direct manipulation of nucleolar size or phase state. Dissecting the precise changes in nucleolar phase behavior during aging, and their effects across different cell types, is ongoing work that may reveal novel targets for pro-longevity interventions. One intriguing possibility is the development of drugs that directly target nucleolar phase behavior to “rejuvenate” the nucleolus in aged cells. Although therapeutics that modulate the condensation state of membraneless organelles remain in their infancy, the nucleolus stands out as a strong candidate for intervention.

## Data Availability

Not applicable.
